# Magnetic resonance neurography appearance and diagnostic evaluation of peripheral nerve sheath tumors

**DOI:** 10.1038/s41598-019-43450-w

**Published:** 2019-05-06

**Authors:** Heng Zhai, Yinzhang Lv, Xiangquan Kong, Xi Liu, Dingxi Liu

**Affiliations:** 1 Department of Neurology, Union Hospital, Tongji Medical College, Huazhong University of Science and Technology, Wuhan, 430022 China; 2 Department of Radiology, Tongji Hospital, Tongji Medical College, Huazhong University of Science and Technology, Wuhan, 430030 China; 3 Department of Radiology, Union Hospital, Tongji Medical College, Huazhong University of Science and Technology, Wuhan, 430022 China

**Keywords:** Surgical oncology, Peripheral neuropathies

## Abstract

Imaging appearances of peripheral nerve sheath tumors by MRI are difficult distinguish from soft-tissue tumors. The objective of this study was to evaluate the feasibility and imaging appearance of high-resolution 3-T magnetic resonance neurography (MRN) of the diagnosis of peripheral nerve sheath tumors (PNSTs) using sampling perfection with application-optimized contrasts using different flip angle evolution (SPACE) sequences. We retrospectively evaluated the MRI and 3D Short tau inversion recovery sampling perfection with application-optimized contrasts using varying flip-angle evolutions (3D-STIR SPACE) sequences of 30 patients with PNSTs diagnosed by surgery and pathology. The contrast-enhanced 3D-STIR SPACE images were retrospectively analyzed and evaluated for the visualization of PNSTs. The tumors were evaluated by their number, location, morphology, size, signal intensity and enhancement characteristics. The imaging findings and characteristic signs of conventional MRI scanning and contrast-enhanced 3D-STIR SPACE sequences were compared. In these cases, conventional MRI images display the location, number, shape, size and signal characteristics of the lesions. These tumors were mostly solitary and had a well-defined boundary. Compared to conventional MRI images, imaging appearances including neurogenic origin, length of the peripheral nerves and relation to the nerve of PNSTs on 3D-STIR SPACE images were more accuracy (P < 0.05). Compared to 3D-STIR SPACE images, contrast-enhanced images can more clearly display background suppression of the peripheral nerves. The “split fat” sign and “target” sign were seen in some patients. 3D STIR SPACE sequences demonstrate its significant capacity to diagnostic evaluate and location of PNSTs. This article comprehensively reviews radiologic findings and illustrates the MRN features of PNSTs. 3D-STIR SPACE sequences be used for preoperative evaluation of PNSTs.

## Introduction

Nerve sheath tumors are relatively common neurogenic tumors arising from nerve sheath cells. They usually grow slowly, present as a solitary mass and affect the peripheral, cranial or sympathetic nerves^1–3^. The common types of peripheral nerve sheath tumors (PNSTs) include schwannomas, neurofibromas and malignant peripheral nerve sheath tumors (MPNSTs). Most of these tumors, such as schwannomas and neurofibromas, are benign upon histological examination^1,2,4^. In addition to these benign tumors, some malignant lesions exist such as MPNSTs. MPNSTs are rare neurosarcomas with a low incidence and ^4^poor prognosis^2,4,5^.

Schwannomas and neurofibromas have more complete epineurium around them because they arise from nerve sheath cells and are separated from the involved nerves. These two types of tumors share some magnetic resonance imaging (MRI) features. Malignant nerve sheath tumors, because of an incomplete envelope, are more likely to invade the surrounding tissue^6,7^. PNSTs are difficult to diagnose at onset, as they are not easy to detect. After the PNSTs are clearly diagnosed, they can be removed surgically while retaining nerve function^1,8,9^.

MRI has many advantages, such as being multidimensional, providing multiple sequences and having good soft-tissue resolution. It allows non-invasive and early identification of PNSTs^10–12^. 3-T MRI provide higher spatial resolution and thinner slices with improved contrast-to-noise ratio, which is particularly useful for imaging of peripheral nerves^13–15^. Three-dimensional (3D) acquisitions combine with high T2-weighted sequences are used to image the spinal nerves and can provide high resolution image quality in peripheral nerve diseases^15,16^. The 3D T2-weighted SPACE sequences have been performed in the brachial plexus and lumbar-sacral plexus in patients with spinal disease^13,17^.

High-resolution 3-T MR neurography (MRN) is an effective technique for examining peripheral nerves. For conventional MRI images of the PNSTs, it is not possible to visualize the relationship between tumor lesions and involved nerves^11,15^. MRN can clearly show pathological and normal tissue characteristics and is the technology modality used to evaluate PNSTs because it has obvious advantages in diagnosing PNSTs and evaluating their relationship with the surrounding tissue structure^16,18^. In recent years, because of the ever-increasing use of MRN combined with contrast-enhanced scanning and a variety of post-processing techniques, MRN can be used to display wide-range, high-resolution and the course of peripheral nerves^4,15,19^. MRN is the major investigative tool that can be used to identify nerve abnormalities caused by PNSTs. It has unique advantages in the diagnosis of PNSTs and also be used for presurgical localization.

In our research, we reviewed and analyzed MRI conventional scanning imaging and neuroimaging of thirty cases of PNSTs confirmed by postoperative pathology or puncture biopsy. Then, we summarized the PNST characteristics, which showed clear and definite radiologic findings of MRN that can be used to diagnose PNSTs.

## Materials and Methods

### Clinical data

This study was a retrospective analysis of patient data. We collected the clinical data from 30 patients with PNSTs in the Union Hospital Affiliated with Tongji Medical College of the Hua Zhong University of Science and Technology. Of these PNSTs, 29 were benign, confirmed as schwannomas via pathology, and 1 was malignant. All cases were ultimately confirmed by pathology or puncture biopsy after surgery. The age range of the patients was 15~52 years (mean age 38 ± 10 years), including 12 males and 18 females. No patients or their family members had a history of neurogenic tumors. The clinical symptoms of the patients included limb numbness, regional pain, an enlarged mass, lumbar discomfort and skeletal muscle weakness (Table 1).

**Table 1:** Clinical data of PNSTs.

Characteristic	
Age	15~52 (mean age 38 ± 10)
**Gender**	
Male	12
Female	18
**Origin**	
Right brachial plexus	4
Left brachial plexus	9
Right lumbar plexus	6
Left lumbar plexus	3
Right sacral plexus	4
Left sacral plexus	4
Mean size	4.3 ± 1.7 cm (range from 2.3 to 12.1 cm)
**Symptom**	
Minor motor dysfunction	11
Lump	6
Sensory disturbances	23
Others	3
**Shape**	
Spindle	12
Oval	13
Irregular	5

#### Magnetic resonance neuroimaging

We used a super high-field superconducting 3.0 Tesla (Magnetom Trio, Siemens, Erlangen, Germany) MRI scanner to obtain thin-section planar scans (1 mm section thickness). Written informed consent was obtained from each participant and the participant’s primary parent before participation. The consent of the parent has been obtained before the scanning the subject under the age of 18. The retrospective study was approved by the Ethics Review Committee at Tongji Medical College, Huazhong University of Science and Technology. All procedures were performed in accordance with the relevant guidelines/regulations in the Declaration of Helsinki.

Every patient underwent MRI scanning of the location of the tumor lesions. A spinal matrix coil and a neck matrix coil or a body matrix coil were selected, and patients were instructed to breathe calmly. The MRI protocol included coronal T1-weighted and T2-weighted images (T1WI and T2WI, respectively), fat-suppression short T1 inversion recovery (STIR), and 3D-STIR SPACE images. All examinations required contrast-enhanced scanning with Gd-DTPA (gadopentetate acid) intravenous administration. The MRN protocol used in our study is outlined in Table 2.

**Table 2:** 3T conventional and MRN examination protocol for evaluation of the Peripheral nerve.

	FOV (mm)	Section Thickness (mm)	TR/TE (ms)	Matrix
**MR imaging sequence**				
Coronal T1WI	320–448	5	600/20	320 × 320
Coronal T2WI-FS	320–448	5	4000/39	320 × 320
Axial T2WI	320–448	5	4000/39	320 × 320
Axial T1WI	320–448	5	600/20	320 × 320
Axial T2WI-FS	320–448	5	4000/39	320 × 320
3D-STIR SPACE	384–448	1	4000/286	448 × 448
3D-STIR SPACE-with contrast-enhanced	384–448	1	4000/286	448 × 448
Coronal T1WI-with contrast-enhanced	320–448	5	600/20	320 × 320
Axial T1WI-with contrast-enhanced	320–448	5	600/20	320 × 320
**Additional arm examination if desired**				
Coronal T2WI	320–448	5	4000/39	320 × 320
sagittal T2WI	320–448	5	4000/39	320 × 320
Coronal T1WI-FS-with contrast-enhanced	320–448	5	600/20	320 × 320

Note: FS = fat suppression; contrast-enhanced = Gd-DTPA 0.2 ml/kg I.V.

#### Image post-processing technology analysis

After magnetic resonance neuroimaging sequence scanning and contrast-enhanced 3D-STIR SPACE scanning, the original images were post-processed using the maximum intensity projection (MIP), curved planner reconstruction (CPR) and multi-planner reconstruction (MPR). The original images and post-processing images were independently analyzed by two radiologists who had 10 and 20 years of experience in MRI. By analyzing the images from the conventional MRI, 3D-STIR SPACE sequences and contrast-enhanced 3D-STIR SPACE sequences, we performed a comprehensive evaluation of the displayed lesions and the relationships between the lesions and nerves.

#### Statistical analysis

General clinical data are expressed as the means ± standard deviation. Statistical analysis was performed with SPSS software (version 23.0). A chi-squared test was used to compare imaging manifestations of PNSTs between MRI and MRN. A P-value < 0.05 was considered to indicate a statistically significant difference.

## Results

### MRI appearances

**Figure 1:**
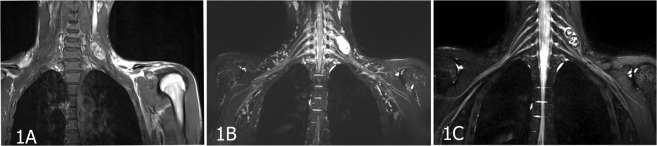
An 18-year-old male patient with peripheral nerve sheath tumors in the left C6 spinal nerve. (**A**) The lesion in the left neck was well circumscribed with inhomogeneous enhancement on coronal enhanced T1WI. (**B**) Coronal MIP 3D-STIR SPACE image shows inhomogeneously enhanced mass closely related to the left C6 nerve and pushing the left C5 nerve. (**C**) Compared with T1WI image, contrast-enhanced 3D-STIR SPACE image has better effect of background suppression, the mass was originated from C6 nerve.

**Figure 2:**
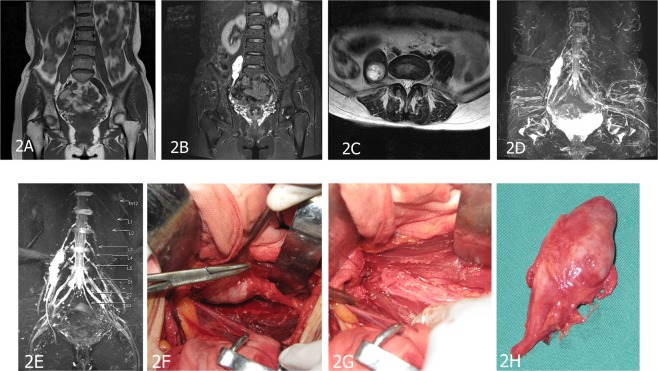
A 47-year-old female patient with peripheral nerve sheath tumors in the right psoas. (**A**) Coronal T1-weighted image (T1WI) shows an isointense signal mass and were not clear among the boundary of adjacent tissues. (**B**) In the coronal T2-weighted fat-suppressed imaging sequence (T2WI-FS), the tumor displays hyperintense signal with a well-defined edge. (**C**) The tumor appeared to be clearly inhomogeneously enhanced in axial-enhanced T1WI. (**D**,**E**) Contrast-enhanced 3D-STIR SPACE image with maximum intensity projection shows that the tumor originated from the right L3 spinal nerve. (**F**–**H**) Surgical view and macroscopic specimen of nerve sheath tumor.

**Figure 3:**
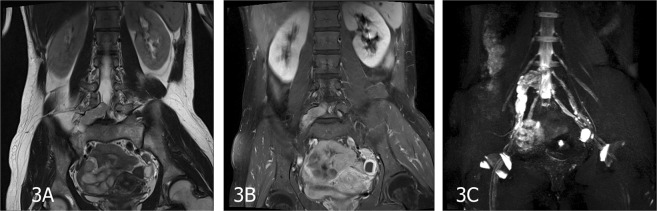
A 36-year-old female patient with a peripheral nerve sheath tumor in the right L5 spinal nerve. (**A**) On coronal T2-weighted images (T2WI), the mass shows short signal with ill-defined and irregular shape. (**B**) The tumor appears as a slightly hyperintense signal and was obviously enhanced on coronal enhanced T1-weighted fat-suppressed images. (**C**) Contrast-enhanced 3D-STIR SPACE with MPR image, the tumor was derived from the right L5 nerve, involving the lumbosacral trunk and from location along nerve distribution.

**Figure 4:**
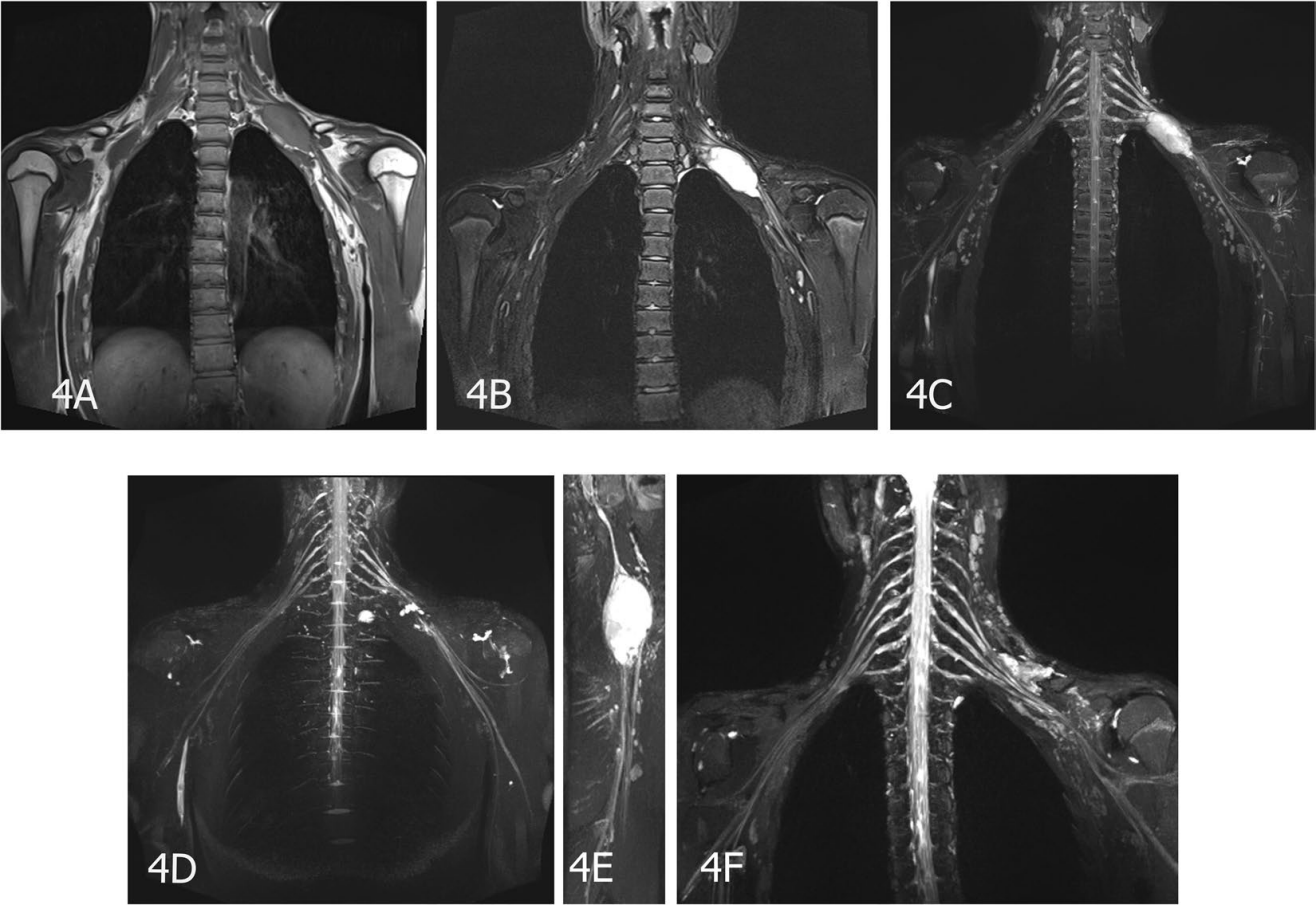
A 15-year-old male patient with an MPNST in the left C5 spinal nerve. (**A**) Coronal T1-weighted images (T1WI), isointense signal with ill-defined mass located in the left supraclavicular region. (**B**) On coronal T2-weighted fat-suppressed imaging image (T2WI-FS), the lesion shows hyperintense signal with well-defined shape. (**C**–**E**) 3D-STIR SPACE and enhanced images show that the tumor was originated from the left brachial plexus. (**F**) Enhanced 3D-STIR SPACE image was received after postoperative examination.

The PNSTs were mainly located in the intermuscular space of the neck, extremities, paraspinal region, and retroperitoneal region. Among the 29 patients with benign peripheral nerve sheath tumors (BPNSTs), 12 patients had a tumor in the brachial plexus and its branches (Fig. 1), 9 patients had a tumor in the lumbar plexus and its branches (Fig. 2A–E), and 8 patients had a tumor in the sacral plexus and its branches (Fig. 3A–C). The single case of an MPNST was located in the brachial plexus (Fig. 4A–D).

Except for 1 patient who showed a long group of lesions along the left lower limb, the remaining tumors were solitary and well defined in T2WI. The most common shapes of lesions were fusiform, oval, beaded, round, fusiform or beaded, and compared to the nerve distribution, the tumors were oriented along their long axis. The size of the PNSTs varied, with the largest size reaching 12.1 cm and the smallest size only 2–3 cm (range from 2.3 to 12.1 cm, 4.3 ± 1.7 cm). On T1WI, tumors appeared as isointense or low signals, and the image signal of the lesion was essentially uniform without a well-defined edge. On T2WI, the tumors appeared as hyperintense or slightly hyperintense signals with well-defined edges, and the imaging signal of the lesion was inhomogeneous. The tumor appeared obviously inhomogeneously enhanced, and the edges showed obvious intense signals; some lesions in the central part of the tumor showed multiple intensities.

It is important to visualize common morphological signs in neurogenic tumors. The typical signs include target signs, entering and exiting nerve signs, fat tail signs and split-fat signs. The “target sign” shows high signal intensity in the periphery and low signal intensity in the central portion on T2WI. A “split fat sign” depicts the well-defined tumor surrounded by a layer of fat on longitudinal T1WI. In our study, we observed “split-fat signs” (Fig. 2A) and “target signs” (Fig. 1C).

MRN appearances: All the PNSTs originated from a single nerve branch.Among these cases, 4 had PNSTs in the right brachial plexus, 9 had PNSTs in the left brachial plexus (Fig. 1), 1 had an MPNST in the left brachial plexus (Fig. 4), 6 had PNSTs in the right lumbar plexus (Fig. 2), 3 had PNSTs in the left lumbar plexus, 4 had PNSTs in the right sacral plexus (Fig. 3), and 4 had PNSTs in the left sacral plexus (Table 1). Among the 29 cases with BPNSTs, one originated from the left sciatic nerve, starting from the initial portion of the left sciatic nerve, distributed along the trunk and branches of the left sciatic nerve, and fully distributed in the left sciatic nerve and its main branches at the left ankle level. The other BPNST cases originated from a single nerve segment.

Except the one PNST that involved the whole length of the left sciatic nerve and its branches, all the other PNSTs involved one segment of the origin nerve. The images clearly displayed the uninvolved proximal and distal segments of the tumor lesion, and some nerves near the tumors were slightly swollen (Figs 1, 2), along with the distal part of the lesion in normal nerves. The tumors appeared to exhibit a pushing phenomenon on the peripheral nerve. For example, the tumors that originated in the left cervical 6 spinal nerve pushed into the left cervical 5 spinal nerve (Fig. 1).

Compared with conventional MR imaging, conventional and contrast-enhanced 3D-STIR SPACE scanning can directly display the nerve structure itself, which has an important clinical value in diagnosing the origination nerve and the scope of the involved nerves in PNSTs (Table 3). When comparing conventional and contrast-enhanced 3D-STIR SPACE scanning images, the tissues containing rich water in the lymph nodes and small veins in the background of conventional 3D-STIR SPACE scanning images appeared as hyperintense signals; the lesion signal was obviously reduced in the contrast-enhanced images, the background suppression effects were improved, and the ganglion showed low signal levels, which looked to be a filling defect. Contrast-enhanced 3D-STIR SPACE scanning can clearly display the relationship between the tumor and the adjacent nerve with reduced interference and provide more helpful information for PNST diagnoses (Figs 1C, 2D,E, 3C).

**Table 3:** Comparison of imaging manifestations between MRI and MRN.

		Conventional MRI scan	Conventional MRN	Contrast-enhanced MRN	Conventional MRI scan VS Conventional MRN		Conventional MRN VS Contrast-enhanced MRN	
					Pearson Chi-Square	p value	Pearson Chi-Square	p value
Tumor characteristic	present	30	30	29				
	absent	0	0	1	equal	~	1.017	p = 1
The origin of tumor	present	4	27	27				
	absent	26	3	3	35.306	p = 0.00	equal	~
Invasion	present	1	27	27				
	absent	29	3	3	45.268	p = 0.00	equal	~
Pushing phenomenon	present	4	27	26				
	absent	26	3	4	35.306	p = 0.00	0.162	p = 1
Background suppression	present	0	7	28				
	absent	30	23	2	7.925	p = 0.011	30.24	p = 0.00

## Discussion

PNSTs are relatively rare soft-tissue tumors that are usually classified into two categories in clinical practice: BPNSTs and MPNSTs. The major types of BPNSTs include schwannomas and neurofibromas^2,7,20^. Primary peripheral neurogenic tumors include schwannomas, neurofibromas, and malignant nerve sheath tumors, which can also be part of neurofibromatosis. Secondary tumors include metastatic tumors and peripheral tissue tumors around the nerve^7^.

Schwannomas and neurofibromas are benign tumors with mild clinical symptoms. Most patients experience minor motor dysfunction symptoms when at the hospital. These patients are characterized by sensory dysfunction, local swelling and soreness, and radiating pain in the affected area of innervation. As in the above case that involved the whole length of the left sciatic nerve, the patient moves freely, only feeling numbness and a sense of electric shock occasionally in the left lower limb. In terms of the location of the tumors, all the tumors in this study were located in the brachial plexus, the lumbar plexus and the sacral plexus. Symptoms are presumed to be related to the sensory disturbances in these areas. Surgical resection and radiotherapy are the main therapeutic strategies for these tumors^8^.

Peripheral nerves have complex anatomical structures and adjacent structures and are tortuous. Ultrasound, X-ray myelography, computed tomography (CT), computed tomography myelography (CTM), and conventional and contrast-enhanced MRI have their advantages and disadvantages, but these techniques cannot completely meet the requirements for the clinical diagnosis of peripheral nerve tumors^6,10,18^. Magnetic resonance imaging (MRI) is a non-invasive technology used for the diagnosis of PNSTs, they also have common imaging features.

MRN was first proposed by Filler *et al*. in^21^, and the development of MRN continues to progress. This technique mainly includes diffusion-weighted imaging, heavy T2 fat-suppression imaging and PROSET imaging^17,22–24^. Some scholars have assessed the use of diffusion tensor imaging (DTI) but have reported that it is not mature enough at the moment for spinal nerve imaging^25^. Compared to conventional MRI scanning, magnetic resonance neurography (MRN) can directly display the full length of a nerve and its invasion by a PNST in addition to showing intraneural and extraneural lesions^19,26^.

In this study, we used STIR fat-suppression, three-dimensional high-resolution imaging, heavy T2 hydrography, and sensitivity encoding (SENSE) parallel acquisition methods in combination with 3D-STIR SPACE technology. The advantages of this process are mainly shown in the following three aspects: (1) The larger imaging range, where images can be scanned up to 448 mm * 448 mm. The brachial plexus can be visualized to the elbow joint or even farther, and the lumbosacral plexus can be visualized to the level of the knee-joint or even farther. (2) The fat-suppression effect, which allows for more homogeneous and stable images. (3) The higher spatial resolution, allowing spatial resolutions smaller than 1-mm voxels^27^.

The disadvantages of this method are that the background suppression effect is not good, and the characteristics of tumors in heavy T2, short TI and fat-suppression sequences that determine its imaging range appear as hyperintense signals, which affects observations, especially in the tissues such as lymph nodes and small blood vessels with slow blood flow containing an abundant amount of water.

For this reason, we added Gd-DTPA enhancement to the 3D-STIR SPACE sequence scans in this study. We found that contrast-enhanced scans significantly improved the background suppression effect and increased the contrast-noise ratio, thus displaying the length, continuity and morphology of the spinal nerve more prominently. These enhanced images can clearly show the spinal nerve branch and main branches, except in thick nerves such as the radial nerve, ulnar nerve, median nerve, sciatic nerve, and femoral nerve. These images can also show the axillary nerve, musculocutaneous nerve, obturator nerve and other relatively small nerves. Small branches, such as the superior scapular nerve and the superior gluteal nerve, can be clearly displayed in some volunteers.

3D-FIESTA-C (three-dimensional fast imaging employing steady state acquisition with cycled phases) and IDEAL (least-squares estimation) sequences are commonly used to directly display small nerves or nerve root filaments^28^. Conventional imaging is essential for the selection of sequences. The selection of conventional or contrast-enhanced scanning in 3D-STIR SPACE sequences depends on the location of the tumor.

Numerous neuropathies involve the ganglion, such as tumors, trauma, and inflammation. We should perform MRN conventional scanning prior to contrast-enhanced MRN imaging, otherwise the imaging of tumor lesions will not be sufficient, and these lesions will appear only as low-intensity signals that look like a “negative shadow “.

For patients with tumors in the spinal nerve fiber area, it is important to directly visualize the tumor origin from the spinal nerve by using MRN. MRN can clearly define the range of the tumor’s involvement with the nerve. If the tumors do not originate from the nerve, MRN can also show whether the nerve has been invaded or pushed by tumors and the relationship between the tumor and surrounding tissue. These radiological findings are important for the selection of surgical approaches and tumor surgical methods. MRN is used to identify the nerve origin of a tumor and the extent of its involvement before surgery. Therefore, both the surgical field and the traumatic effects of surgery can be reduced^29^. In some patients, MRN was performed before and after the operation (Fig. 4). The scope of the lesion was clearly defined, which has great potential in aiding surgeons during the operation. In our study we demonstrated feasible method to aid diagnoses and for guiding the choice of surgical treatments in patients with tumors (Figs 2F–H, 4F).

## Conclusion

3D-STIR SPACE sequences and contrast-enhanced sequences can show the relationship between tumors and the spinal nerves. These techniques can clearly demonstrate whether the tumor originated from a certain segment of a certain area and whether other spinal nerves are involved; therefore, we can infer whether the pathology of the tumor is benign or malignant. With the lack of a “dumbbell-shaped” performance guide for simple extraspinal neurogenic tumors, it is difficult to judge whether the tumor originates from the nerve, blood vessel, or muscle when using conventional examinations. Neuroimaging is highly valuable in estimating whether the tumor originates from the nerve.

In conclusion, MRI conventional sequences and MRN can clearly display the appearance of PNSTs. In particular, contrast-enhanced 3D STIR-SPACE imaging can clearly show important information about a tumor’s nerve origin, involvement extent and the relationship with the adjacent nerve. This technique will be of great clinical value for aiding early diagnoses and will provide guidance for the surgical treatment of PNSTs.
